# Harnessing shrimp shell waste: enhanced chitinase production through optimization techniques for ecofriendly solutions using bacteria

**DOI:** 10.1186/s13068-026-02808-9

**Published:** 2026-09-22

**Authors:** Basma T. Abd-Elhalim, Mona A. Ashour

**Affiliations:** Department of Agriculture Microbiology, Faculty of Agriculture, Ain Shams University, Shubra El-Khaimah, Cairo, 11241 Egypt

**Keywords:** Chitinase, Central composite design, Cytotoxicity activity, *Priestia megaterium*, Partial purification, Plackett–Burman design, Shrimp shell waste

## Abstract

Chitinous wastes, particularly shrimp shell residues, represent a major underutilized biomass resource with significant potential for bioconversion into high-value products. In this study, four thermophilic bacterial strains (*Bacillus amyloliquefaciens* BT 2022, *Bacillus licheniformis* Basma87, *Priestia megaterium* AMD 2024, and *Streptomyces maritimus* MSQ-2021) were screened for their ability to produce chitinase using shrimp shell waste (SSW) as the sole carbon and nitrogen source. Among these, *P. megaterium* AMD 2024 exhibited the highest chitinolytic activity. Optimization of fermentation parameters was initially performed using the one-variable-at-a-time (OVAT) approach, identifying 5% SSW, 60° C, 72 h incubation, pH 7.0, 200 rpm shaking, and 2% inoculum size as optimal conditions, yielding 93.24 U/mL chitinase activity. Subsequent statistical optimization using response surface methodology (RSM) with Plackett–Burman design (PBD) and central composite design (CCD) significantly enhanced enzyme production, achieving maximum activity of 273.3 U/mL, representing a 2.93-fold increase compared to OVAT. Partial purification via ammonium sulphate saturation (60–80%) resulted in 260.0 U/mL activity with 95.13% relative activity. Cytotoxicity assays using normal human skin cell line HFB4 confirmed the biocompatibility of the purified enzyme at concentrations up to 260.0 U/mL. These findings demonstrate that shrimp shell waste can be efficiently valorized into chitinase through microbial fermentation, providing an eco-friendly solution for waste management and offering safe, sustainable applications in agriculture, industry, and cosmetics.

**Graphical Abstract:**

**Figure:**
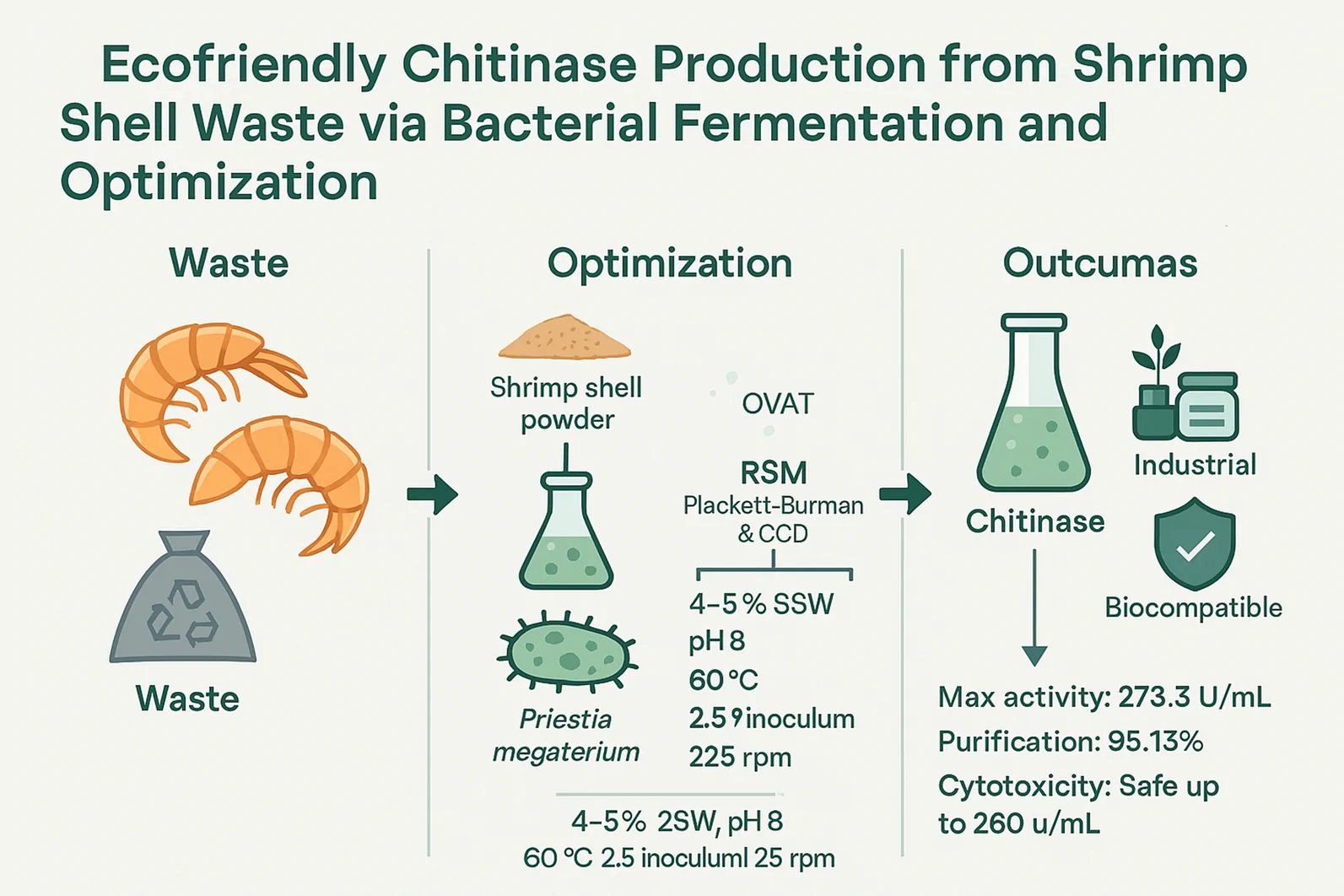

## Introduction

The aquatic biosphere is a major contributor to global chitin production, generating approximately 1011 tons annually, primarily from crab, shrimp, and lobster shells [1–3]. Despite this abundance, an estimated 6–8 million tons of chitinous waste are discarded worldwide, often buried in landfills or dumped into marine environments, leading to environmental concerns [3, 4]. Shrimp shells, which typically contain chitin, protein, calcium carbonate, and lipids, represent a valuable raw material for bioconversion into eco-friendly products such as fertilizers, animal feed, and enzymes [4–6].

Recent advances have emphasized recycling these wastes into high-value bioproducts, including biodegradable fertilizers, animal feeds, and enzyme-based biopesticides [5, 6]. Among these, chitinase (EC 3.2.1.14) is particularly important due to its ability to hydrolyze chitin into oligomeric and monomeric components such as N-acetyl-D-glucosamine [3, 7, 8].

Chitinases are widely distributed across bacteria, fungi, insects, mammals, and higher plants, and their properties vary depending on the source organism. Extremophilic microorganisms, including thermophiles, halophiles, and acidophiles, are especially valuable for producing chitinases with novel characteristics [9–11]. Applications of chitinase span agriculture, food safety, pharmaceuticals, textiles, and cosmetics. They serve as biocontrol agents against phytopathogens, enhance food shelf life, and contribute to the production of N-acetyl-D-glucosamine and other bioactive compounds [12–14].

Efficient enzyme production, however, depends on optimizing fermentation conditions such as substrate concentration, pH, temperature, inoculum size, and agitation speed [15, 16]. Submerged fermentation (SmF) has proven advantageous over solid-state fermentation (SsF), offering reduced contamination risk and improved control of nutrient and environmental parameters [17].

In this context, the present study investigates four bacterial strains—*Bacillus amyloliquefaciens* BT 2022, *B. licheniformis* Basma87, *Priestia megaterium* AMD 2024, and *Streptomyces maritimus* MSQ-2021—for their ability to produce chitinase using shrimp shell waste as the sole carbon and nitrogen source. By employing both one-variable-at-a-time (OVAT) and response surface methodology (RSM), we aim to maximize chitinase yield under controlled fermentation conditions. The outcomes of this research not only enhance enzyme production efficiency but also advance waste valorization strategies, contributing to sustainable agriculture and industrial biotechnology.

## Materials and methods

### Chemicals used

Chitin powder was obtained from Sigma, Aldrich. Glucose oxidase peroxidase kits were purchased from (BIO-ADWIC) El Nasr pharmaceutical chemicals Co. (Egypt). Nutrient broth (NB) and MGPY (malt extract 1%, glucose 1%, yeast extract 0.3%, peptone 1%) media were purchased from OXOID, India. All chemicals used in this study are fine analytical grades.

### Shrimp shell waste collection (SSW)

Shrimp shell waste (SSW) was collected from a local fish market in Cairo, Egypt. It was used for chitinase fermentation and optimization investigations. The chemical composition of the shrimp shell was as follows: calcium carbonate (40%), protein (35%), chitin (40%), and lipids (8%) as reported by the Central Laboratory of the Agriculture Research Centre, Giza, Egypt.

### Bacteria strains

Four thermophilic bacteria strains of *Bacillus* *amyloliqueficiens* BT 2022*, B. licheniformis* Basma87*, Priestia megaterium* AMD 2024*, and Streptomyces maritimus* MSQ-2021 were obtained from the culture stock at the Agricultural Microbiology Department, Faculty of Agriculture, Ain Shams University, Cairo, Egypt. Inoculating a loop of freshly made microbial culture into the sterile NB and MGPY media produced the standard inocula for all strains [18] in a 100 mL flask for *Bacillus* spp., *P. megaterium*, and *Streptomyces* sp., respectively. The inoculated flasks were then incubated for 24 h at 50° C and 150 rpm, and O.D. was observed at 620 nm. Each 1 mL of the inoculum contained 2.30–2.74×10^6^ CFU/mL for *Bacillus* spp. and *P. megaterium* strains and 1.5×10^7^ CFU/mL for *S. maritimus*.

### Preparation of shrimp shell waste (SSW)

The SSW was collected, cleaned with tap water, and then dried in a hot air oven for 24 h at 60° C until a constant weight was achieved. The dried SSW was grounded into a fine powder using a laboratory grinder (Moulnix, France). To ensure uniformity in the measurements, the grounded SSW was sieved to eliminate larger particles, resulting in a homogeneous sample size.

### Colloidal chitin preparation

Colloidal chitin was made from chitin powder to be utilized as a substrate [19]. To put it briefly, 40 g of SSW powder gradually added to 600 mL of concentrated HCl, and the mixture was vigorously stirred for 60 min at 30° C until it turned translucent. To remove any accumulated contaminants and undissolved particles on the filter paper, the suspension was filtered and collected. In order to wash the filter, it was suspended in around 2.0 L of cooled Distilled water (DW) and stirred for 30 min, or until the pH reached 5–6. After that, it was kept for a whole day at 4° C in the dark without being stirred. In order to be used as a substrate for the chitinase enzyme for activity assessment, 10 mL of the loose colloidal chitin were dried at 80° C for 24 h after the previous treatment.

### Chitinase production by submerged fermentation technique (SmF)

Chitinase fermentation was performed in shrimp shell waste (SSW) broth (250 mL flasks that contain 5 g of the SSW as the sole carbon and nitrogen source) in 100 mL of tap water [20]. Then inoculated with 2% inoculum and incubated at 50° C for 72 h at 150 rpm on a rotary shaking incubator (Shin Saeng; South Korea). After 15 min of chilling following incubation, the cultures were centrifuged at 10,000 rpm for 10 min using a SIGMA 2–16 P centrifuge. The crude enzyme was then utilized for an evaluation of chitinase activity.

### Estimation of chitinase activity

Chitinase activity was estimated as follows with modification [21]; 2.5 mL of phosphate buffer (0.05 M, pH 7.0), 2.5 mL of 1.0% colloidal chitin, and 0.5 mL of crude enzyme (supernatant) made up the reaction mixture. The tubes underwent one hour of incubation at 50° C. 3.0 mL of HCl (1.0 N) was added to stop the reaction, and it was centrifuged for 10 min at 4° C at 10,000 rpm. One mL of the resultant mixture and one mL of glucose kits were mixed together and incubated for 20 min at 37° C. To lessen the amount of sugars released, the mixture's absorbance was determined using spectrophotometer at 540 nm. U/mL was the measurement of the chitinase enzyme’s activity. The amount of reduced sugar generated in 30 min was used to calculate enzyme activity using the following formula: The glucose standard curve was used to estimate the reduced sugar. Chitinase activity is calculated according to the following Eq. (1) [22]:

1 $$ Enzymeactivity\,\,{U \mathord{\left/ {\vphantom {U {ml}}} \right. \kern-\nulldelimiterspace} {ml}} = (Amount\,\,of\,\,reducing\,\,sugar\, \times \,1000 \times Dilution\,\,factor)/\left( {Molecular\,\,weight\,\,of\,\,gluose \times Time \times Enzyme\,\,volume} \right) $$

One unit of chitinase activity was defined as the amount of enzyme that liberates reducing sugar per min per mL.

A reaction blank containing crude enzyme extract without colloidal chitin was included to determine the background level of reducing sugars present in the enzyme preparation. The absorbance obtained from this blank was subtracted from the test samples, and the corrected reducing sugar values were used for calculating chitinase activity.

### One variable at a time (OVAT) optimization of chitinase production

#### SSW (substrate concentration)

SSW concentration was examined at varying concentrations (5, 10, 15, and 20% w/v) and incubated for 72 h at 150 rpm at 50° C.

#### Effect of temperature on chitinase production

The inoculation media were cultured for 72 h at 150 rpm at various incubation temperatures, ranging from 30 to 70° C with a fluctuation of 10° C, in an effort to ascertain the ideal temperature for chitinase production.

#### Effect of incubation time on chitinase production

In a rotary shaker incubator, an inoculated medium containing 2% inoculum with the ideal SSW concentration and temperature was incubated at 150 rpm. After being incubated for 6, 12, 24, 48, 72, and 96 h, the samples were taken out and centrifuged for 15 min at 10,000 rpm.

#### Effect of pH on chitinase production

The effect of pH level on chitinase synthesis was examined using a digital pH meter (Hanna, Model: HI2210, UK). Using NaOH 1.0 M and HCl 1.0 M at pH levels 4–9 at 1-level intervals, the production medium's pH was adjusted before sterilizing while preserving the ideal SSW concentration, temperature, and incubation time.

#### Effect of shaking speed on chitinase production

With intervals of 50 rpm, the fermentation medium was incubated in 100 mL Erlenmeyer flasks at various shaking speeds in a rotary shaker incubator (Shin Saeng; South Korea) while maintaining optimal SSW concentration, temperature, pH, and incubation time.

#### Effect of inoculum size on chitinase production

Optimal inoculum size was detected by incubating the fermentation medium in 100 mL Erlenmeyer flasks with different inoculum sizes varied between 1 and 4% in a rotary shaker with the suitable temperature, pH, SSW conc., shaking speed, and incubation time. The supernatant was used for chitinase activity assay in each factor investigation.

### Optimization of chitinase production using response surface methodology (RSM)

#### Plackett–Burman design (PBD)

Plackett–Burman design worked to screen for the most important elements influencing chitinase production. The PBD made use of the statistical software program Design-Expert software 12.0.0 (Stat-Ease, Inc., Minneapolis, MN 55413, USA 2019) to assess the relative significance of nutritional and physical parameters for pioneer strain chitinase production. Eleven (n+1) experiments examined a total of six (*n*) variables, including the only nutritional factor SSW as a source of carbon and nitrogen, and five environmental factors (pH, temperature, inoculum size, incubation period, and shaking speed), along with five dummy variables (which will give a sufficient estimate of the error) [23]. Every experiment was run twice, and the design's reaction was determined by averaging the observations. The ( +) and (–) markers, respectively, indicate the high and low levels at which each variable corresponds. Every column denoted an independent variable, and every row denoted a trial run.

Plackett–Burman's experimental design is predicated on the first-order model, which was identified as follows:

2 Y=B0+ΣBixi

When Y is the main response (chitinase activity), B0 is the model intercept, and Bi is the variable estimates. This formula calculated the influence of each variable:

3 $$E (Xi) = 2 (+\Sigma Mi \_\_\Sigma Mi\_) / N$$

where N is the number of trials in Eq. (2), E (Xi) is the tested variable effect, and Mi+ and Mi− are the enzyme production from trials where the variables (Xi) examined were present at high and low concentrations, respectively.

The significance level (*p*-value) of each concentration effect was ascertained using Student's t-test (Xi) in Eq. (4), and the standard error (SE) of the concentration effect was calculated as a square root of the variance of an effect.

4 t(Xi)=E(Xi)/SE

where E (X_i_) is the variable X_i_ effect.

### Central composite design (CCD)

Following the identification of the critical factors for chitinase synthesis by the pioneer strain using a PBD, the key variables derived from the previous step were optimized using a CCD. Twenty sets of tests (batch experiments), six replications at the central point, and three distinct levels (− 1, 0, and +1) were used to estimate the chosen independent variables. Every variable had a centrally coded value of zero. The statistical software package Design-Expert software 12.0.0 (Stat-Ease, Inc., Minneapolis, MN 55413, USA 2019) was used to evaluate the experimental data. Maximizing the equation inside a specific boundary condition yielded the ideal values of the independent variables that provided a theoretical maximum response in Eq. 5. To create an experimental model that explains the response measured to the independent variables, chitinase activity was used as a response (Y) and the data was subjected to multiple regression analysis. Equation 5, which is a second-order polynomial equation, provided the connection between the result and the independent variables.

5 Yi=b0+b1X1+b2X2+b3X3+b11X12+b22X22+b33X32+b12X1X2+b23X2X3+b13X1X3

where b0 is the offset term, b1, b2, and b3 are linear effects, b11, b22, and b33 are squared effects, b12, b23, and b13 are interaction terms, and Yi is the expected response. For statistical analysis and graph plotting, Design-Expert software 12.0.0 (Stat-Ease, Inc., Minneapolis, MN 55413, USA 2019) was utilized. A *p*-value of 0.05 was used to denote that the results were significant, and Fisher's test was employed to evaluate the impact of independent factors on the response. We used various correlation coefficients (r) and adjusted R^2^ to evaluate the second-order polynomial equation's fitness. These three-dimensional contour graphs demonstrated how the response and the coded parameters interacted. The equation generated by the final quadratic model was solved to determine the optimum sites.

### Partial purification of optimized chitinase using ammonium sulphate

In order to obtain 0–80% saturation at 4° C, different doses of solid ammonium sulphate (NH_4_)_2_SO_4_ were added to a final volume of 1 L chitinase crude enzyme [24]. Following the crude enzyme's immersion in an ice bath, solid ammonium sulphate progressively dissolved, attaining a 20% saturation point at 4° C. After that, it was centrifuged for 10 min at 4° C and 10,000 rpm. We discarded the protein pellets that precipitated. It took additional solid ammonium sulphate to obtain 40% saturation at 4° C in the supernatant. Before centrifuging it as said earlier, it was once again chilled for a night at 4° C. This time, the pellet was gathered, and at 4° C of cooling, more ammonium sulphate was added to the supernatant to reach 60% saturation. It was once more centrifuged and left at 4° C overnight. To get 80% saturation at 4° C, more ammonium sulphate was added to the supernatant after collecting the pellets of the precipitated protein from the previous step. In the same way as previously mentioned, the supernatant was extracted. In one mL of pH 4.7 acetate buffer, each pellet that was collected from each stage of ammonium sulphate saturation was dissolved. After dialyzing the pellets against the same buffer for a whole night at 4° C and twice changing the washing buffer, the surplus ammonium sulfate was eliminated using a Spectra/PorR, VWR 2003 dialysis bag. Total protein and the activity of the enzymes precipitated by ammonium sulphate were determined both before and throughout the dialysis process.

### Total protein determination

The total protein was calculated using bovine serum albumin (BSA) as the reference solution, as per Hartree's [25] instructions. following is the calculation of total protein to determine the specific activity of an enzyme:

6 $$ Specific\,\,activity(U/mg\,protien) = (Activity\,\,of\,\,enzyme(U/ml))/Total\,\,protien(mg/ml)$$

The crude enzyme's activity (control) was assumed to be 100% compared to purified enzyme to calculate the relative activity [26, 27].

7 $$\% Relative \,\,activity = (Activity \,\,of \,\,enzyme (U/ml) )/(Initial \,\,activity\,\, of\,\, enzyme (U/ml)) X100$$

### Cytotoxicity determination using MTT test

The effect of partially purified chitinase in different concentrations ranged between 260.0 and 3.12 U/mL was examined against the viability of skin normal cell line (HFB4) using MTT assay. After the incubation period (24, 48, or 72 h), 20 μL of MTT solution was added to each well containing the cells. The cells were allowed to absorb the MTT solution for 5 h at a controlled temperature (25° C). The medium was then removed, and 100 μL of Dimethyl sulphoxide (DMSO) solution was added to each well. After 15 min of incubation at 25° C, the absorbance was determined with an ELISA reader at a wavelength of 570 nm. The results were analyzed to determine the IC_50_ values. This value represents the concentration of the extract that reduced cell growth by 50% compared to untreated cells. To investigate the morphological changes of HFB4 cell line induced by the partially purified chitinase, approximately 10 μL of culture media were carefully removed from each well using a sampler. These samples were then examined under a light microscope at 40× magnification. The focus was on observing changes in the shape and motility of the HFB4 cell line [28].

Optical density was obtained at 560 nm and subtracted background at 620 nm. Optical density should be directly correlated with cell quantity.

8 $$\% Cell \,\,viability=\frac{Mean Abs control-Mean Abs chitinase}{Mean Abs control} x 100$$

where: Abs absorbance at 560 nm.

### Chitinase production parameters

Productivity and specific enzyme yield (Y_E/X_) were calculated using the following equations according to Pirt [29] and Lawford and Roseau [30]:

9 $$Productivity \,\,(U/ml/h)=Enzyme \,\,activity / Time (h)$$

10 $$Specific \,\,enzyme \,\,yield (YE/X)=Enzyme \,\,activity / (g) cell \,\,mass \,\,formation$$

### Analytical statistics

Statistical analyses using the SPSS statistics software (19 version Stat soft, Inc. 2003) at a 5% level (*P* > 0.05). In order to identify differences in averages between treatments, Duncan [31] recommended the use of analysis of variance (ANOVA). There were three duplicates of each experiment.

## Results

### Chitinase production using SmF technique

**Fig. 1:**
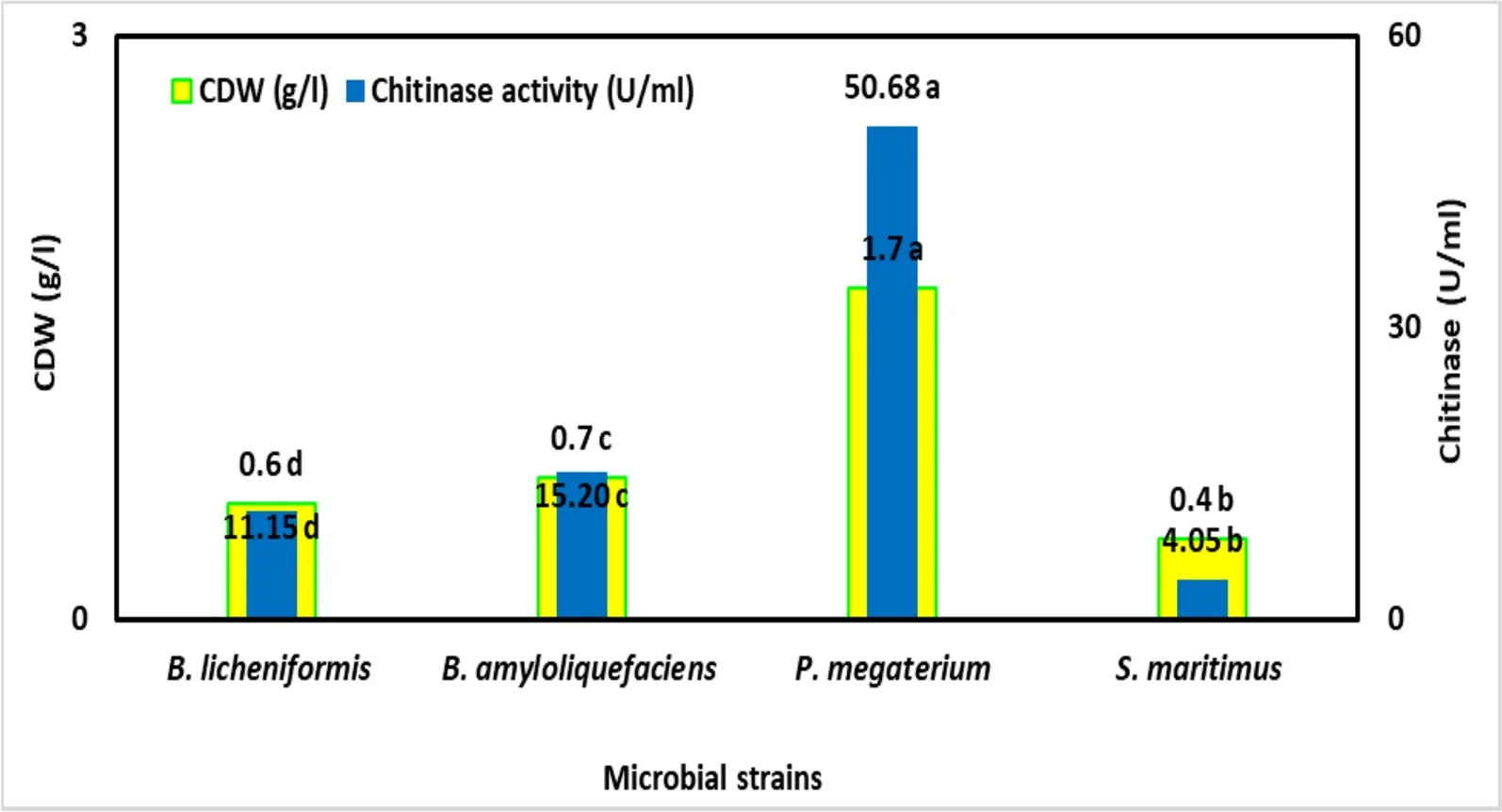
Fermentation of chitinase on SSW broth using various bacteria strains at 50° C after 72 h of incubation at 150 RPM. Variables that are distinguished by the same letter do not differ significantly

Thermophilic bacterial strains of *B. amyloliquefaciens*, *B. licheniformis*, *P. megaterium*, and *S. maritimus* were investigated for chitinase synthesis. The bacterial strains were inoculated in shrimp shell waste (SSW) broth for 48 h of incubation. It was found that *P. megaterium* was the most potent strain with a chitinolytic activity of 50.68 U/mL (Fig. 1). The cell mass (CDW) was estimated at 0.6, 0.7, 1.7, and 0.4 g/L for *B. licheniformis, B. amyloliquefaciens*, *P. megaterium,* and *S. maritimus* after 48 h of incubation, respectively (Fig. 1). For *B. amyloliquefaciens*, *P. megaterium*, and *S. maritimus*, the estimated productivity after 48 h of incubation was 0.23, 0.32, 1.06, and 0.08 U/mL/h, and the specific enzyme yield (Y_E/X_) was 18.76, 20.82, 29.81, and 9.721, respectively. *P. megaterium* was chosen for chitinase production optimization in light of the earlier observations.

### Chitinase production optimization by one variable at a time (OVAT) technique

#### Effect of SSW concentration on chitinase production

**Fig. 2:**
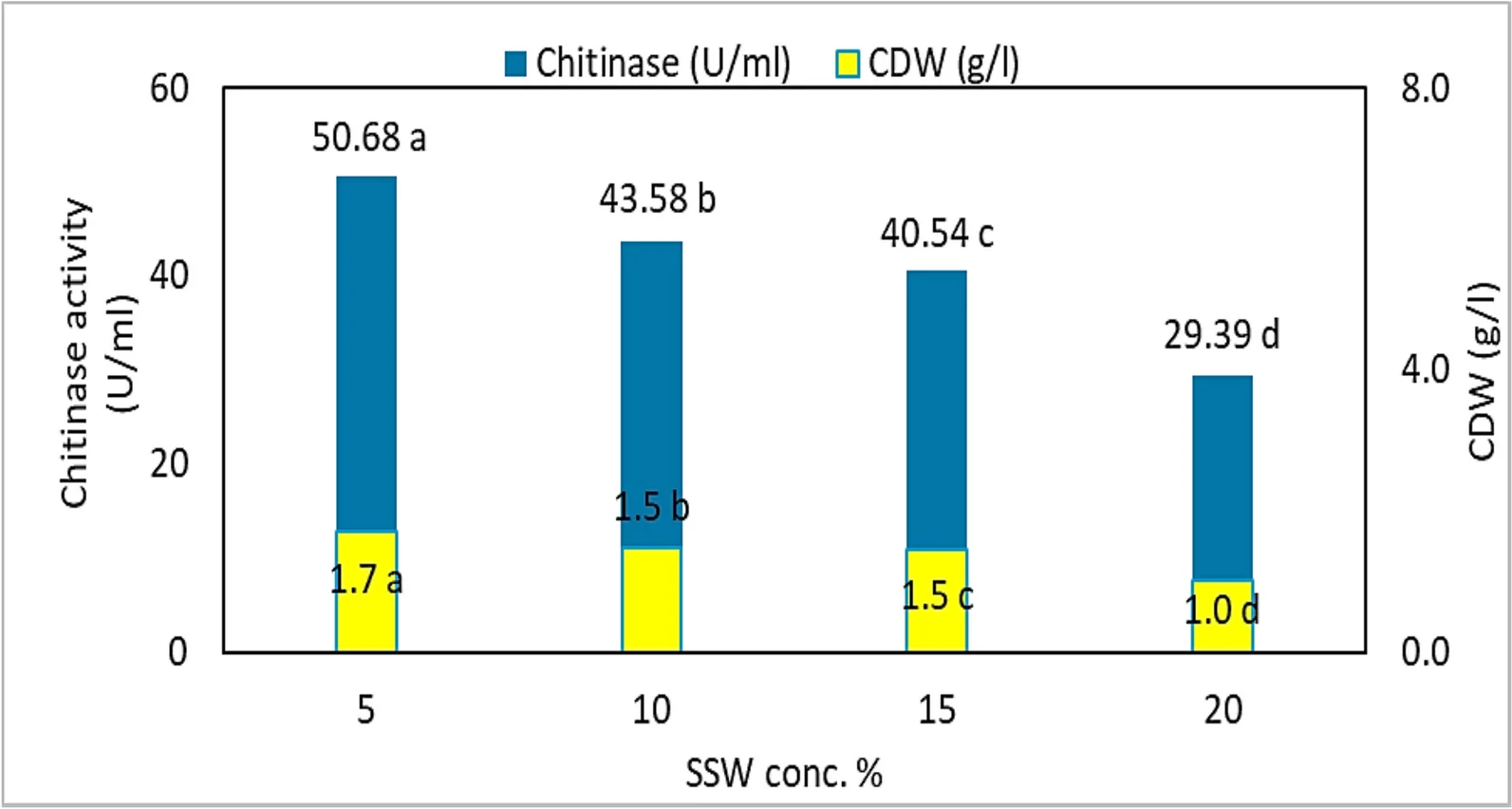
Effect of various shrimp shell waste (SSW) concentrations on chitinase production by *P. megaterium* after 72 h at 50° C at 150 rpm. Variables that are distinguished by the same letter do not differ significantly

To determine the optimal concentration for chitinase synthesis that may be utilized at the industrial level, a range of SSW concentrations, from 5 to 20%, were employed. The findings demonstrated that the pioneer strain *P. megaterium* exhibited the most enzyme activity at 50.86 U/mL at 5% SSW (Fig. 2). The SSW examined concentrations of 5, 10, 15, and 20 g/L, showed 1.7, 2.2, 1.9, and 1.0 g/L of CDW, respectively. At 5, 10, 15, and 20 g/L, the specific enzyme yield (Y_E/X_) was 29.81, 29.71, 27.79, and 29.07, respectively, and the productivity was 1.06, 0.91, 0.84, and 0.61.

#### Effect of temperature on chitinase production

**Fig. 3:**
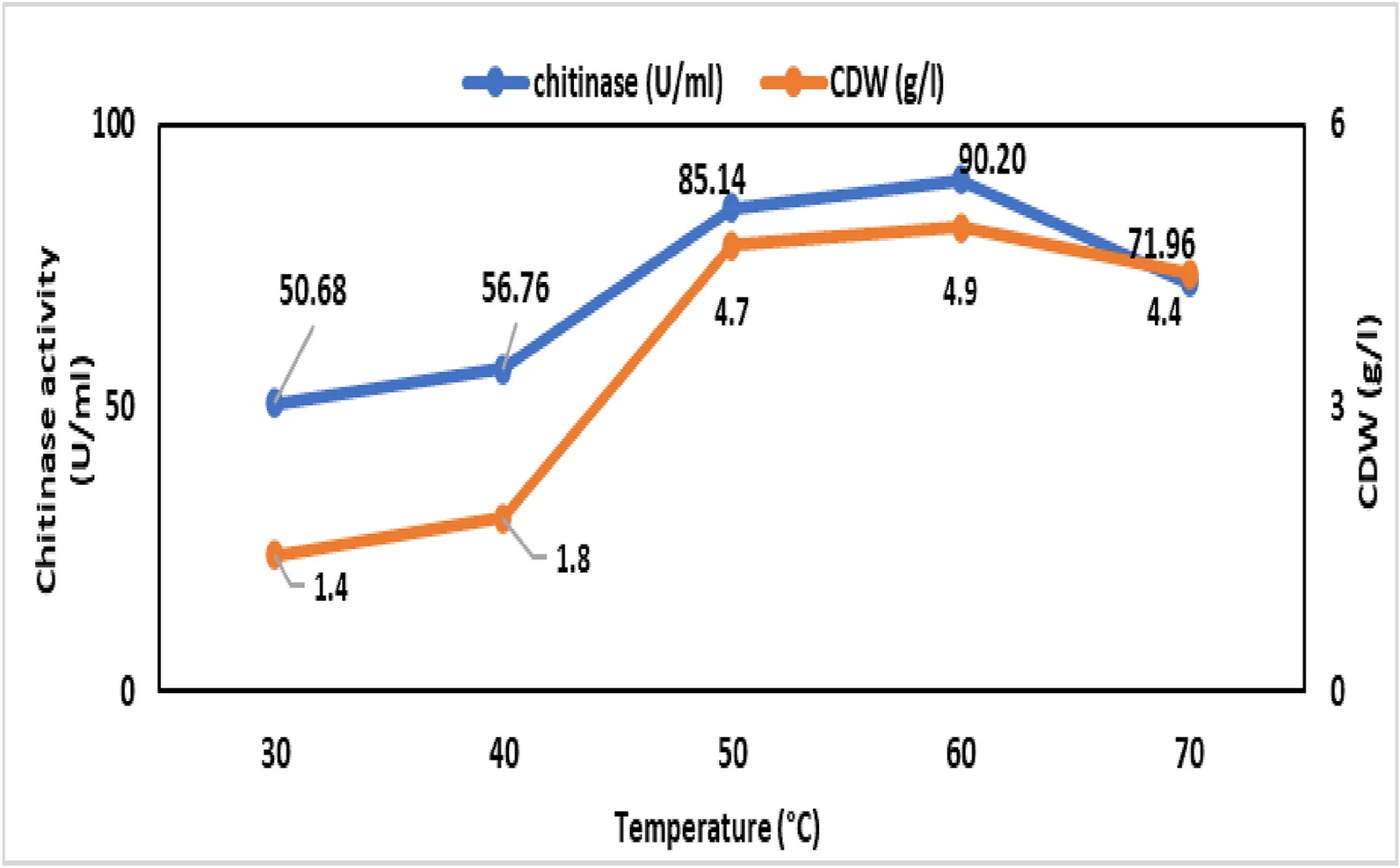
Effect of incubation temperature on chitinase production by *P. megaterium* after 72 h at 150 rpm on SSW broth

Since temperature influences numerous biological processes, changes in the incubation temperature also have an impact on bacterial growth and enzyme synthesis. *P. megaterium* was cultured at 30–70° C to determine the ideal growth temperature for chitinase synthesis. *P. megaterium* produced the most chitinase activity at 60° C, with 90.2 U/mL (Fig. 3). With a temperature of 30, 40, 50, 60, and 70° C, the CDW for *P. megaterium* was 1.4, 1.8, 4.7, 4.9, and 4.4 g/L, respectively.

#### Effect of incubation time on chitinase production

**Fig. 4:**
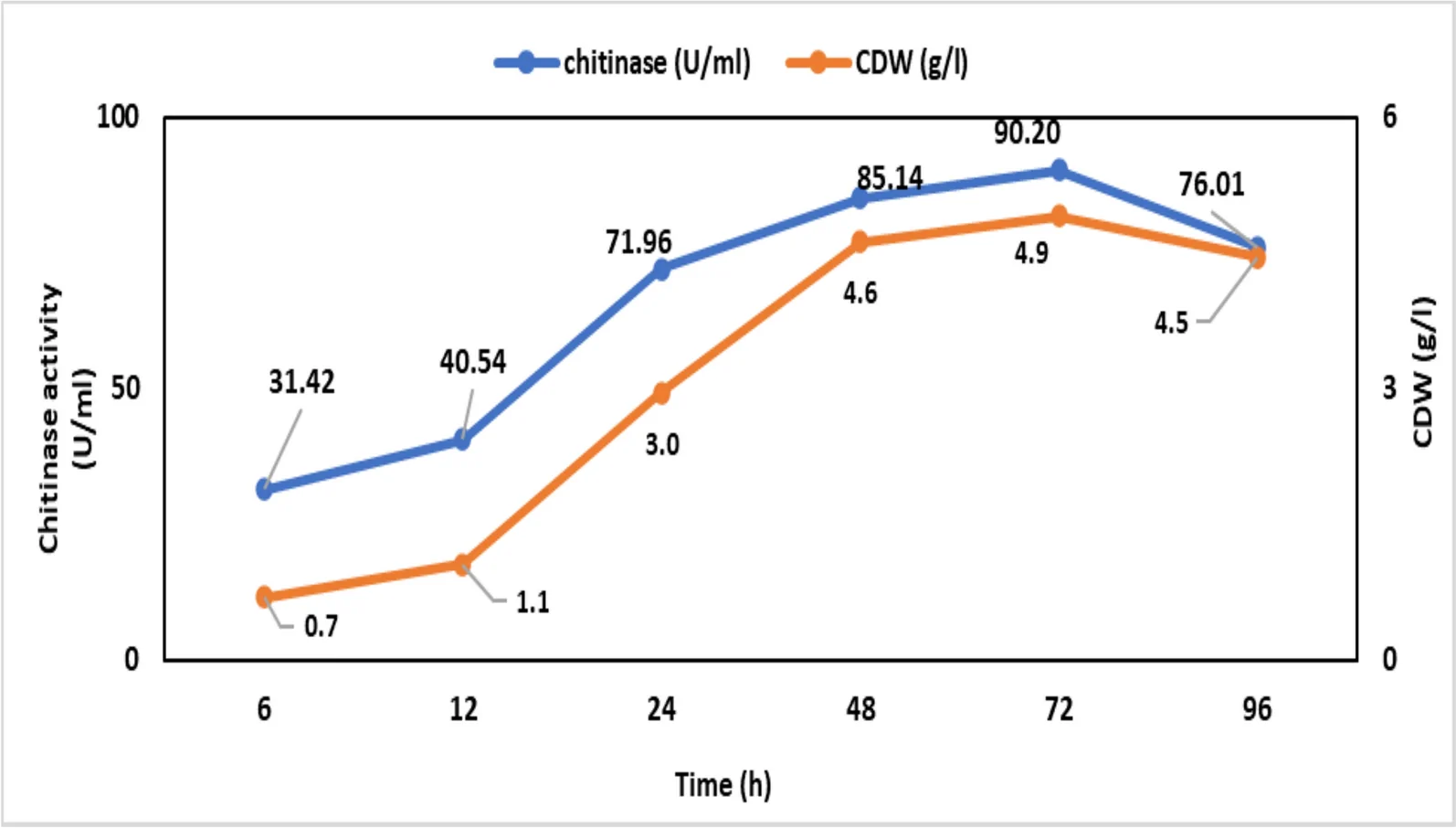
Time kinetics of chitinase production at 60° C by *P. megaterium* at 150 rpm on SSW broth

The chitinase yield peaked at 90.2 U/mL after 72 h of incubation; subsequent production declined, as illustrated in Fig. (4). The cell masses for *P. megaterium* were determined to be 0.7, 1.1, 3.0, 4.6, 4.9, and 4.5 g/L, respectively, after incubation times of 6, 12, 24, 48, and 72 h.

#### Effect of pH on chitinase production

**Fig. 5:**
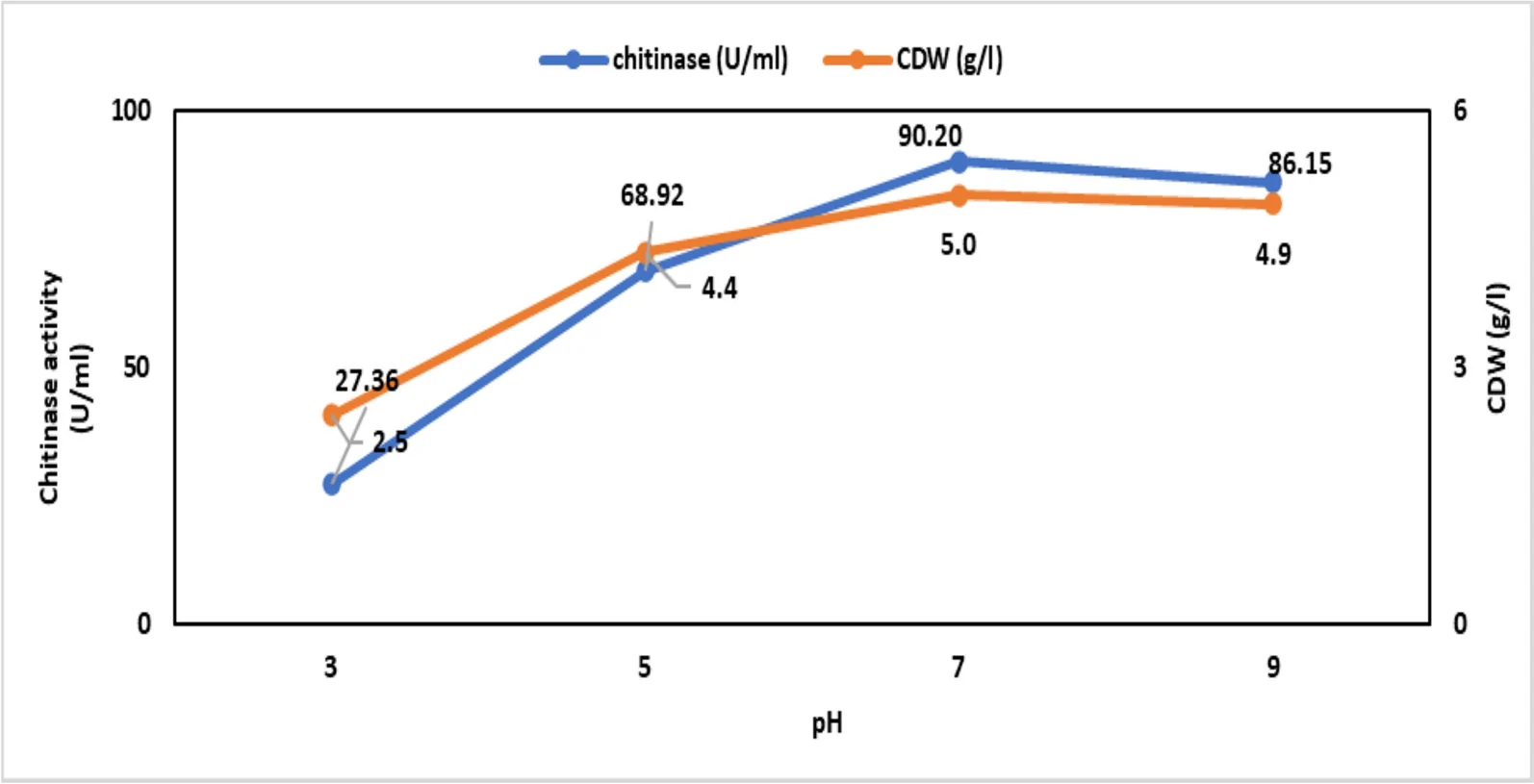
Effect of pH on chitinase production by *P. megaterium* at 60° C after incubation at 150 rpm for 72 h on SSW broth

To determine how pH affected chitinase synthesis*, P. megaterium* was grown at different pH levels (3–9). As seen in Fig. 5, the pH of 7.0 supported the highest chitinase synthesis of any tested pH, at 90.2 U/mL. The cell mass for *P. megaterium* was 2.5, 4.4, 5.0, and 4.9 g/L, with the corresponding incubation pH values being 3, 5, 7, and 9.

#### Effect of shaking speed on chitinase production

**Fig. 6:**
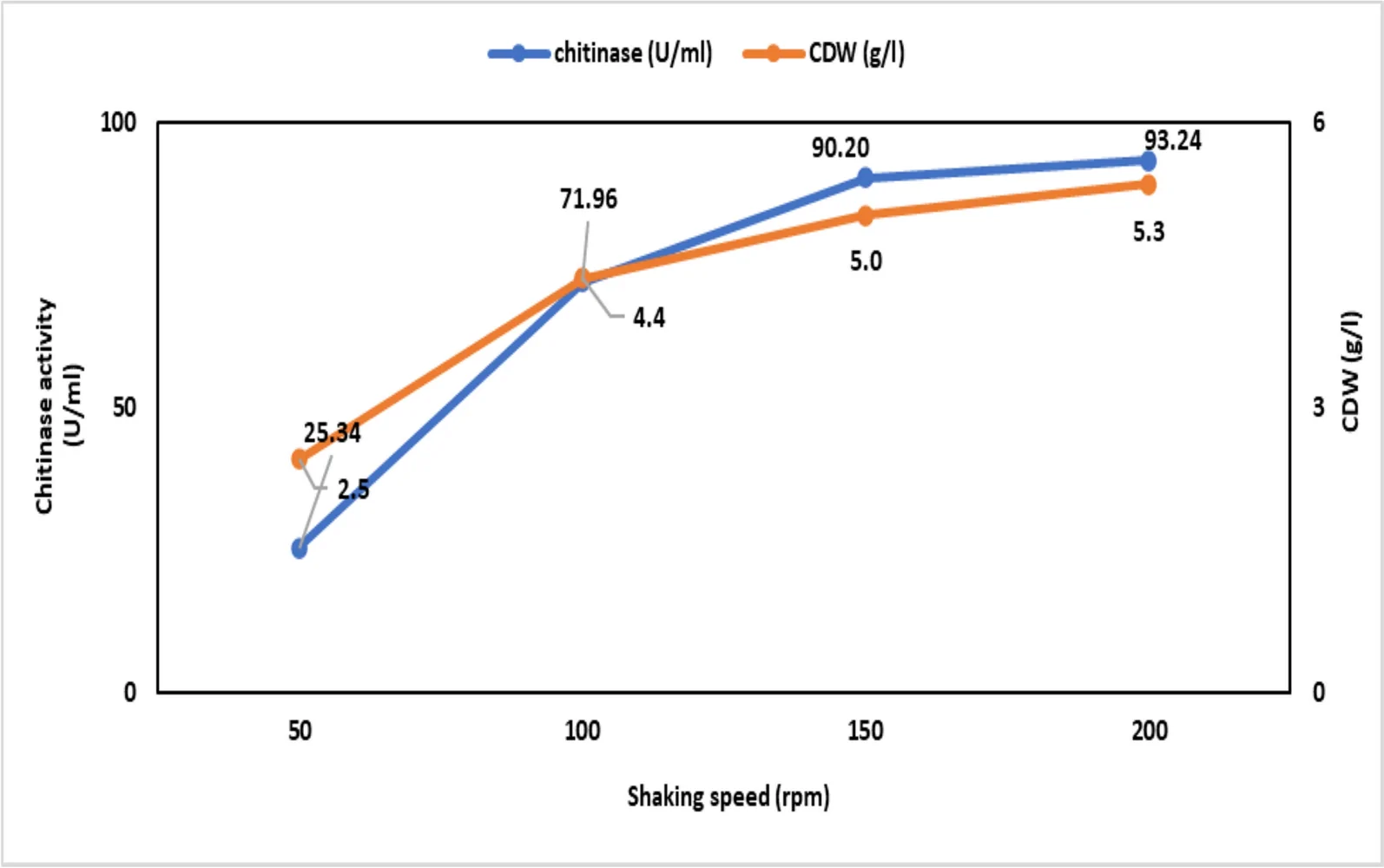
Effect of shaking speed on chitinase production by *P. megaterium* after 72 h at 60° C incubation on SSW broth

Chitinase maximum production with *P. megaterium* under the SmF technique was observed at a shaking speed of 200 rpm (93.24 U/mL) (Fig. 6). The CDW reached 2.5, 4.4, 5.0, and 5.3 g/L with shaking speed of 50, 100, 150, and 200 rpm, respectively.

#### Effect of inoculum size on chitinase production

**Fig. 7:**
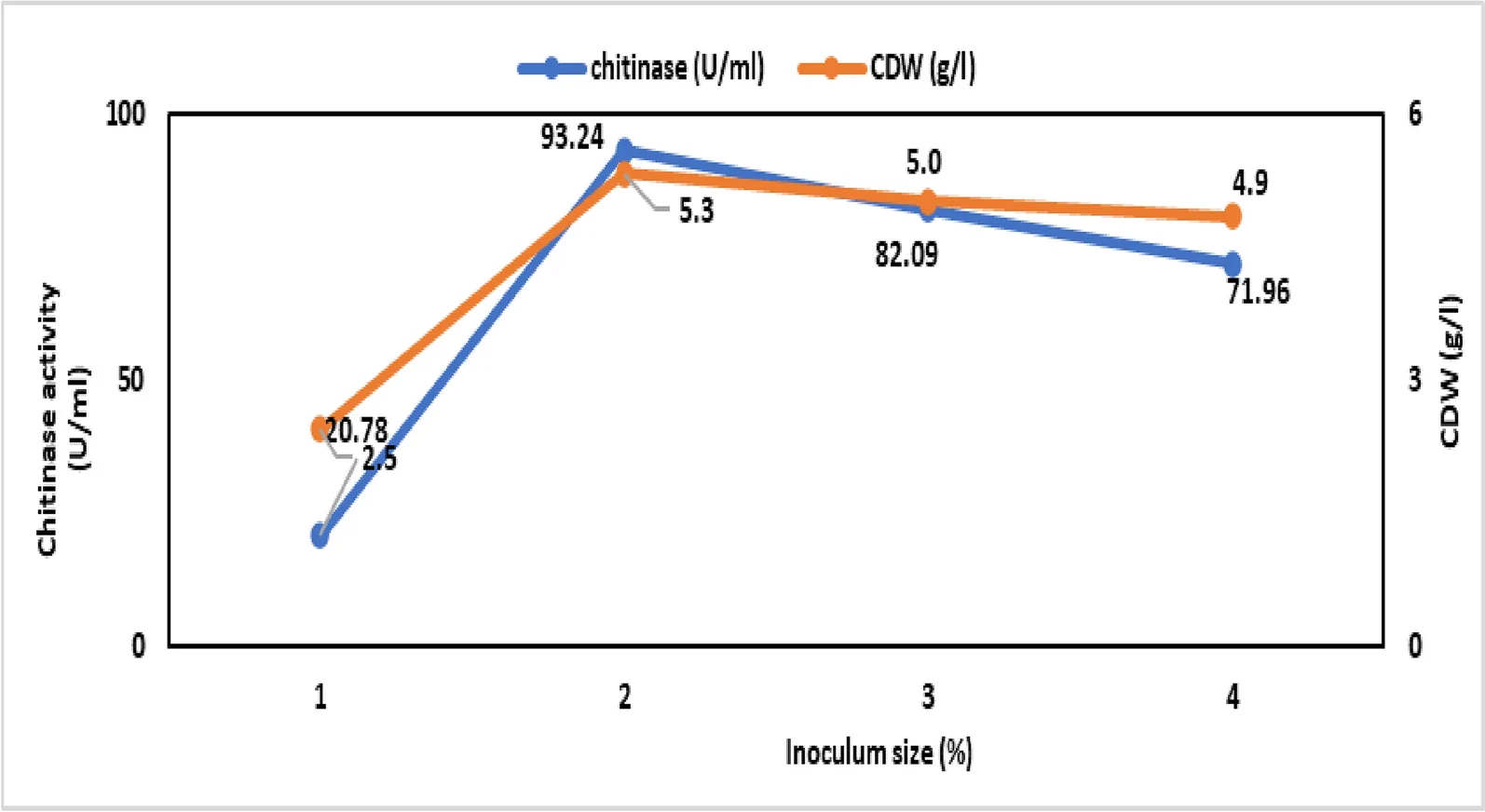
Effect of inoculum size on chitinase production by *P. megaterium* after 60° C incubation for 72 h at 200 rpm SSW broth

Chitinase maximum production with *P. megaterium* under SmF was observed with inoculum size of 2% (93.24 U/mL), then decreased with increased or decreased inoculum volume (Fig. 7). The CDW was achieved at 2.5, 5.3, 5.0, and 4.9 g/L for *P. megaterium*, with inoculum size of 1, 2, 3, and 4%, respectively.

#### Chitinase optimization using Plackett–Burman design (PBD)

The Plackett–Burman experimental design was used to evaluate the effect of independent conditions on *P. megaterium* chitinase production. This optimization method was carried out using six distinct components (variables), as shown in Table (1). These factors were medium constitution SSW (as a single carbon and nitrogen source) and culture conditions (pH, temperature, inoculum size, incubation time, and shaking speed). The design for 12 runs with two levels for each factor. Chitinase activity averages for different trials were given in U/mL. The major influence of each variable on chitinase activity was determined as the difference between both measurement averages made at high (+ 1) and low levels (− 1) of that factor. Data presented in Table (1) showed a wide variation from 265.36 to 122.79 U/mL in 12 experiments due to the strong influence of variables on the activity of chitinase.

**Table 1:** Experimental matrix of Plackett–Burman design for selection of significant variables of chitinase synthesis by *P. megaterium*

Run no	Variables											
	A	B	C	D	E	F	G	H	I	J	K	Chitinase activity (U/mL)
1	5	60	48	9	200	2	1	1	1	–1	–1	265.358
2	5	60	72	9	150	2	–1	1	–1	1	1	222.797
3	10	60	48	9	200	3	–1	–1	–1	1	–1	134.784
4	10	50	48	7	200	2	1	1	–1	1	1	153.625
5	5	50	72	7	200	3	–1	1	1	1	–1	217.922
6	10	50	72	9	200	2	–1	–1	1	–1	1	125.169
7	10	50	72	9	150	3	1	1	–1	–1	–1	162.057
8	10	60	72	7	150	2	–1	–1	1	1	–1	150.726
9	5	50	48	9	150	3	1	–1	1	1	1	138.209
10	10	60	48	7	150	3	–1	1	1	–1	1	147.696
11	5	60	72	7	200	3	1	–1	–1	–1	1	122.794
12	5	50	48	7	150	2	–1	–1	–1	–1	–1	131.095

Variables	Symbol
A B C D E F G H I J K	SSW conc. (%) Temperature (°C) Incubation time (h) pH Shaking speed (rpm) Inoculum size (%) Dummy 1 Dummy 2 Dummy 3 Dummy 4 Dummy 5

To determine the major effect and the significance of each factor, the estimated effects, coefficients mean squares, F-values, and *P*-values for the major effect of each variable are shown in Table (2). The coefficient of determination (R^2^) was 0.999, which indicates a satisfactory representation of the process model and a high correlation between the experimental and predicted values. The following equation was produced for the PBD (first-order model) using Design Expert:

**Table 2:** Analysis of variance (ANOVA) for the selected factorial model of chitinase production by *P. megaterium* by Plackett–Burman design

Variables	Mean square	df	F-Value	p-value (Prob>F)
Model	5201.36	11	352.46	0.0414*
1- SSW conc. (%)	4185.75	1	283.64	0.0378*
2-Temperature (°C)	1122.84	1	76.090	0.0727
3-Incubation time (h)	78.5400	1	5.3200	0.2604
4-pH	8775.93	1	594.68	0.0261*
5-Shaking speed (rpm)	5943.91	1	402.78	0.0317*
6-Inoculum size (%)	8818.74	1	597.58	0.0260*
7-Dummy 1	11,204.5	1	759.25	0.2310
8-Dummy 2	8423.24	1	570.78	0.2660
9-Dummy 3	1543.71	1	104.61	0.6200
10-Dummy 4	1916.53	1	129.87	0.5570
11-Dummy 5	5201.36	1	352.46	0.4140
Std. Dev Mean R^2^	3.840 81.02 0.999			

*Significant at 5% level (*P*≤0.05). df= degree of freedom

11 Ychitinaseactivity=-218.73818-7.4706∗SSWconc.+1.93463∗Temperature-0.21319∗incubationtime+27.04307∗pH+0.89024∗Shakingspeed-54.21791∗Inoculumsize+30.55659∗H+26.49409∗J-11.34206∗K-12.63767∗L

**Fig. 8:**
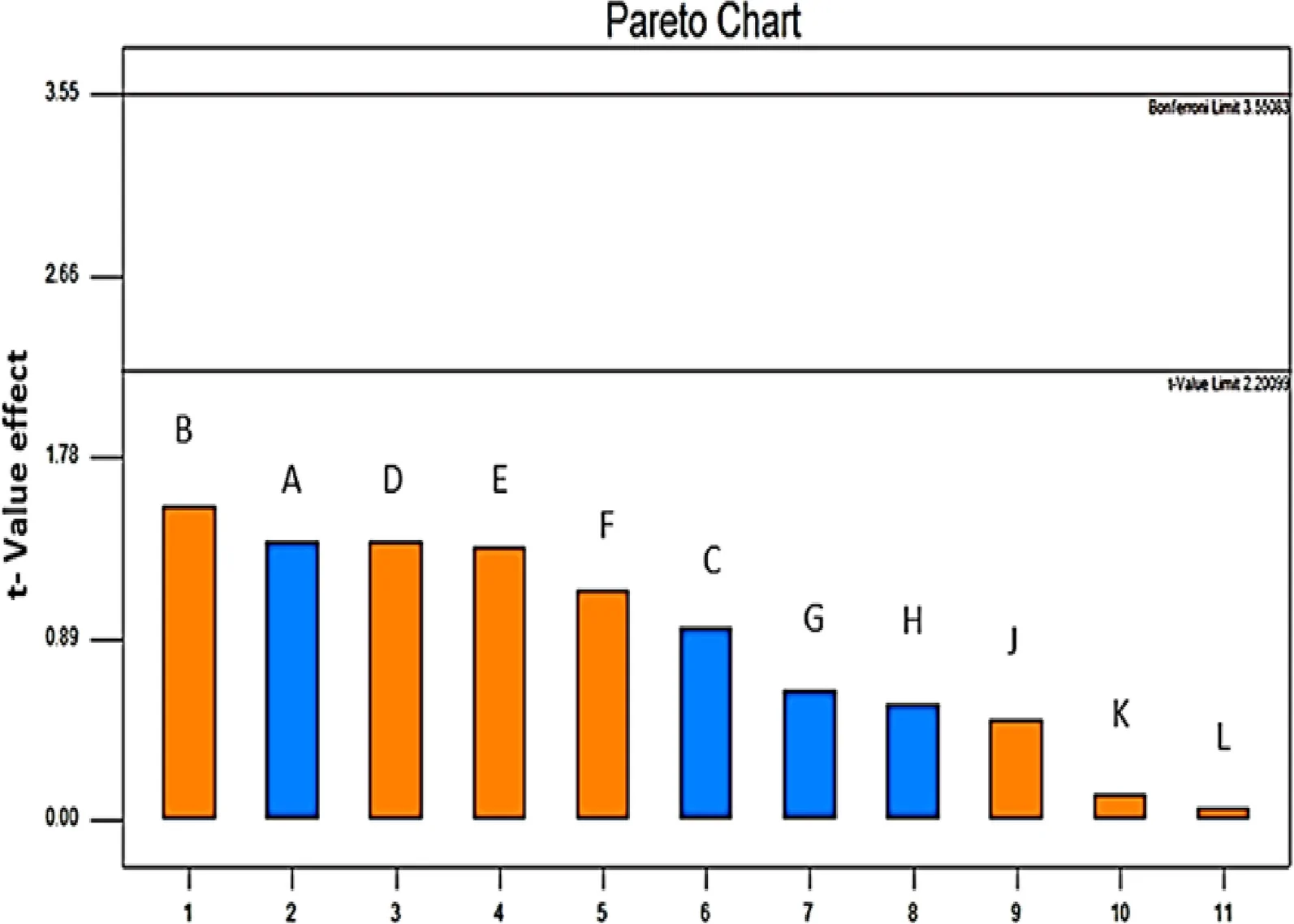
Pareto chart for the selected factorial model of chitinase production by *P. megaterium*. Orange columns for the positive-effect factors and blue for the negative-effect factors

The Pareto chart (Fig. 8) was used to show the effect of all variables on chitinase activity in the PBD. The positive coefficients for all factors suggest a linear effect on the increment in the activity of chitinase, except SSW, conc. (%), incubation time (h), Dummy1, and 2, which had negative coefficients implying a linear effect on the decrement in chitinase activity, respectively. The response surface optimization technique at CCD is used to optimize chitinase production, considering the important elements chosen by the PBD.

### Chitinase optimization using Central composite design (CCD)

**Fig. 9:**
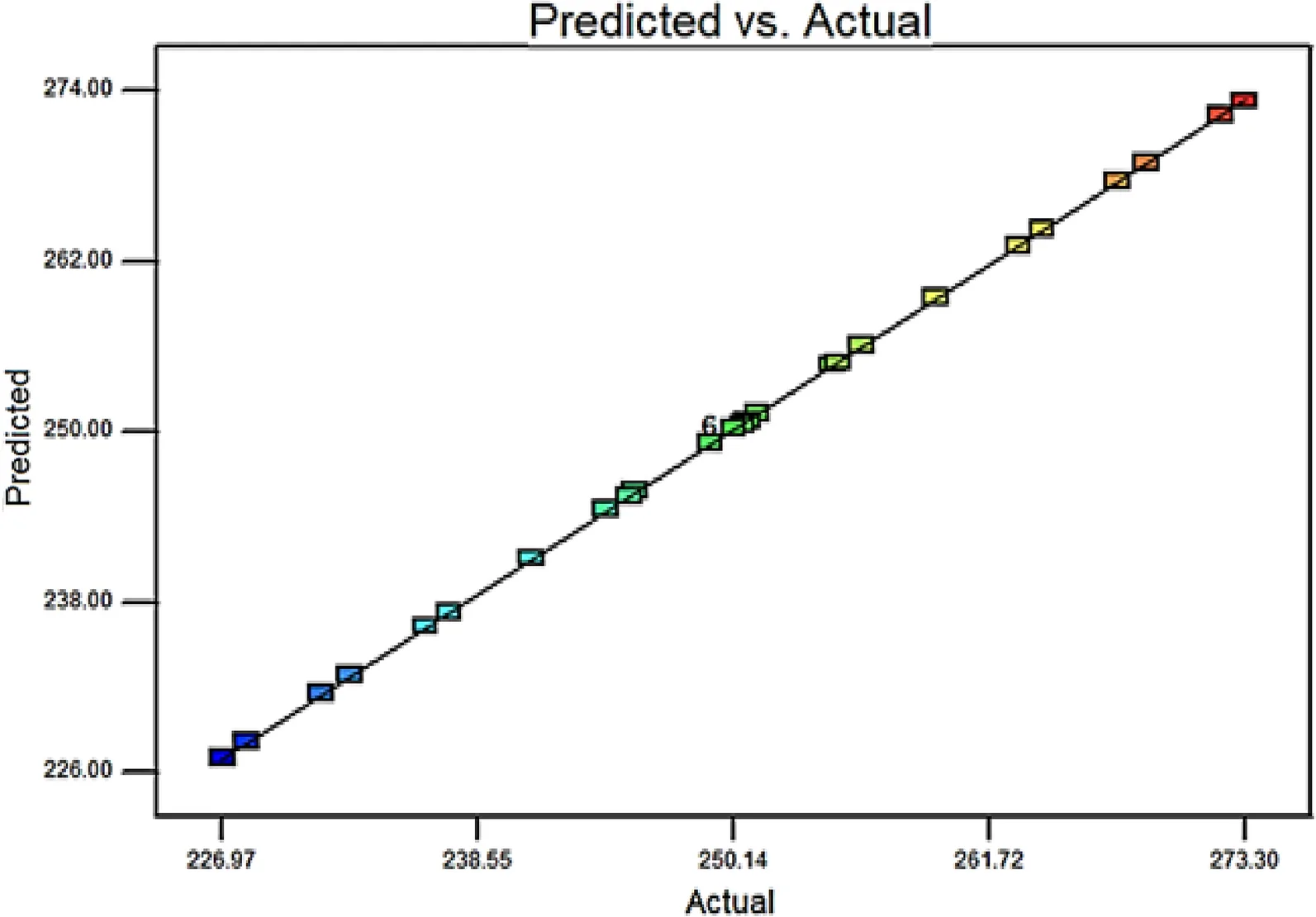
Predicted versus actual results for the selected factorial model of chitinase fermentation by *P. megaterium* by central composite design (CCD)

To ascertain the ideal values and the interactions between the chosen relevant factors, central composite design (CCD) was used. In this study, a total of 30 experiments with a different combination of SSW (A), pH (B), inoculum size (C), and shaking speed (D) were performed and the results of experiments for studying the effects of four independent variables on chitinase activity at three different levels coded as − 1, 0, and +1 are presented along with actual and predicted response (Table 3). The data showed significant variation in the chitinase activity. Run number 2 showed a high chitinase activity of 273.3 U/mL. While the lowest activity was recorded in run number 21 with an activity of 226.972 U/mL. The actual and predicted data for chitinase activity were strongly correlated and revealed to each other, as pointed out in Fig. (9). The response surface quadratic model's F-test and ANOVA were used to assess the model's statistical significance, which is summarized in Table (4).

12 $$Chitinase = + 250.18 - 6.45*A - 11.56*B - 2.36*C - 0.054*D + 0.000* AB + 0.000*A*C + 0.000*A*D + 0.000* B*C + 0.000*B*D + 0.000* C*D + 0.021* A^2 - 0.021* B^2 - 0.021* C^2 + 0.10* D^2 +0.000*A*B*C + 0.000*A*B*D + 0.000*A*C*D + 0.000*B*C*D + 0.000*A^2*B + 0.000*A^2*C + 1.75*A^2*D + 0.000*A*B^2$$

whereas; A= SSW%, B= pH, C= inoculum size%, and D= shaking speed (rpm).

**Table 3:** Central composite design matrix (CCD) of independent variables used in RSM studies for chitinase synthesis by *P. megaterium*

Run order	SSW (%)	pH	Inoculum size (%)	Shaking speed (rpm)	Chitinase (U/mL)
1	4	10	1.5	225	254.889
2	4	8	2.5	225	273.303
3	3	11	2.0	200	245.724
4	3	11	3.0	200	241.013
5	4	10	3.5	225	245.466
6	4	10	2.5	225	250.177
7	5	9	2.0	200	255.950
8	3	11	3.0	250	244.405
9	3	9	3.0	250	267.531
10	5	11	2.0	250	236.217
11	4	10	2.5	225	250.177
12	3	9	2.0	250	272.242
13	5	9	2.0	250	259.342
14	3	9	3.0	200	264.138
15	4	10	2.5	275	250.570
16	5	9	3.0	250	254.631
17	5	9	3.0	200	251.238
18	4	10	2.5	225	250.177
19	3	9	2.0	200	268.850
20	5	11	3.0	250	231.505
21	4	12	2.5	225	226.972
22	4	10	2.5	225	250.177
23	4	10	2.5	225	250.177
24	4	10	2.5	175	250.785
25	6	10	2.5	225	237.278
26	5	11	3.0	200	228.113
27	5	11	2.0	200	232.824
28	2	10	2.5	225	263.077
29	3	11	2.0	250	249.117
30	4	10	2.5	225	250.177

**Table 4:** Analysis of variance (ANOVA) for the selected factorial model of chitinase production by *P. megaterium* by central composite design (CCD)

Variables	Mean square	df	F-Value	*p*-value (Prob>F)
Model	199.40	22	16,747.8	< 0.0001*
A-SSW	332.81	1	27,953.6	< 0.0001*
B-pH	1069.6	1	89,834.9	< 0.0001*
C-Inoculum size	44.390	1	3728.70	< 0.0001*
D-Shaking speed	0.0230	1	1.94000	0.2062
AB	0.0000	1	0.00000	1.0000
AC	0.0000	1	0.00000	1.0000
AD	0.0000	1	0.00000	1.0000
BC	0.0000	1	0.00000	1.0000
BD	0.0000	1	0.00000	1.0000
CD	0.0000	1	0.00000	1.0000
A^^2^	0.0120	1	1.00000	0.3506
B^^2^	0.0120	1	1.00000	0.3506
C^^2^	0.0120	1	1.00000	0.3506
D^^2^	0.3000	1	25.0000	0.0016*
ABC	0.0000	1	0.00000	1.0000
ABD	0.0000	1	0.00000	1.0000
ACD	0.0000	1	0.00000	1.0000
BCD	0.0000	1	0.00000	1.0000
A^^2^B	0.0000	1	0.00000	1.0000
A^^2^C	0.0000	1	0.00000	1.0000
A^^2^D	16.330	1	1371.80	< 0.0001*
AB^^2^	0.0000	1	0.00000	1.0000
Std. Dev Mean R^2^	0.610 236.7 0.999			

*Significant at 5% level (*P*≤0.05). df= degree of freedom

**Fig. 10:**
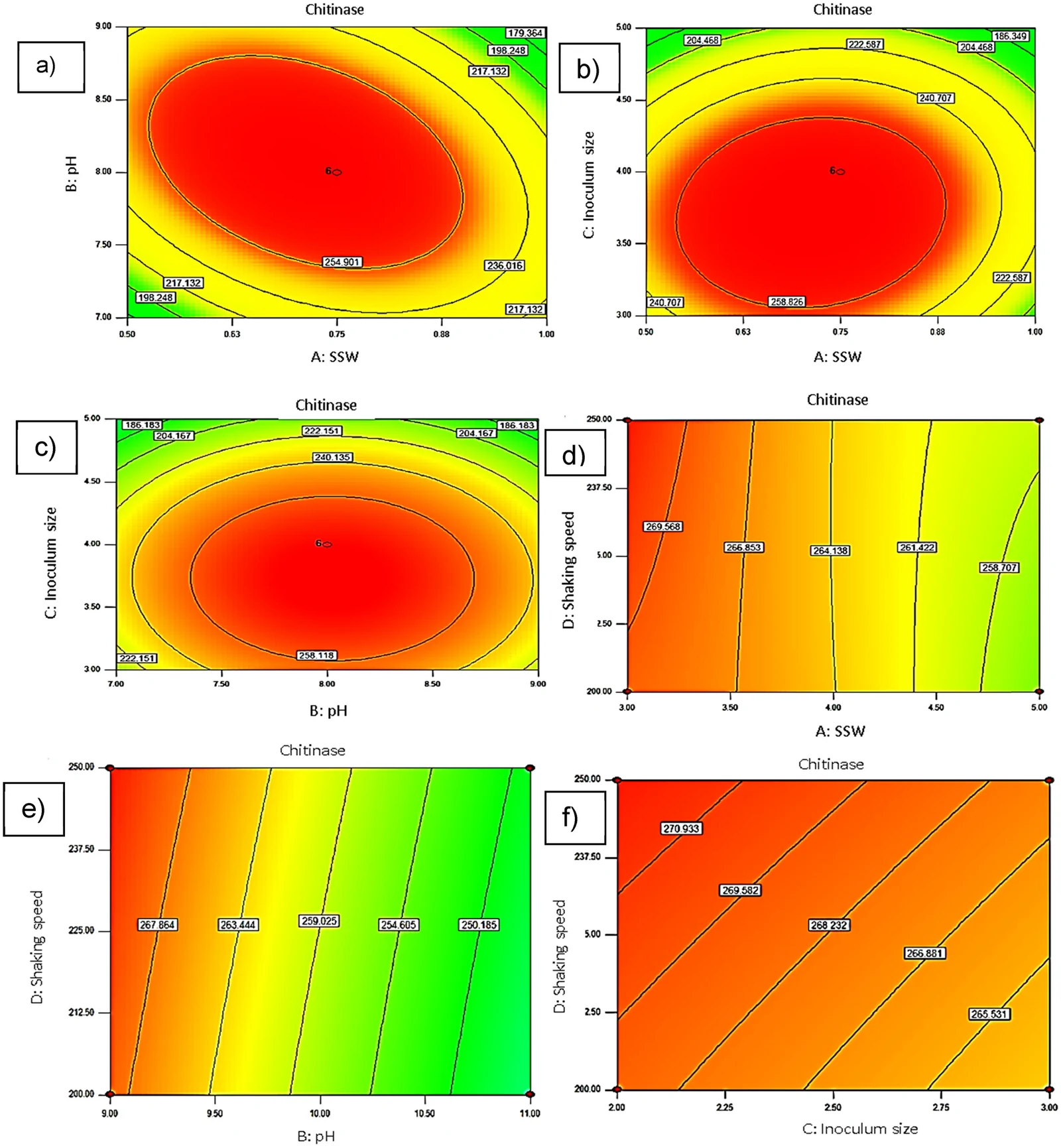
Central composite design (CCD) two-dimensional contour plots for the selected factorial model of chitinase fermentation by *P. megaterium* showing the effect of SSW concentration, pH, inoculum size, and shaking speed and their mutual effect on the chitinase activity (a) SSW *vs.* pH with an inoculum size of 2.5% and shaking speed of 225 rpm (b) SSW *vs.* inoculum size with pH of 8.0 and shaking speed of 225 rpm (c) pH *vs.* inoculum size with SSW concentration at 4% and shaking speed of 225 rpm (d) SSW *vs.* shaking speed with pH 8.0 and 2.5% of inoculum size (e) pH *vs.* shaking speed with SSW concentration at 4% and 2.5% of inoculum size (f) Shaking speed vs. inoculum size with SSW concentration at 4% and pH 8.0

Two-dimensional contour plots are graphical based on the model equation to explain the interaction among variables and to determine the optimum level of each factor for chitinase activity (Fig. 10a–f). Each figure presents the effect of two factors, while the other factor was held at a zero level [32, 33]. The maximum predicted value is indicated by the surface confined to the smallest ellipse in the contour diagram. Elliptical contours are obtained when there is a perfect interaction between the independent variables [10, 34]. The coordinates of the central point within the highest contour levels in these figures represent the optimum condition and concentrations of respective variables [32].

Chitinase production is predicted to be increased with a moderate increase in concentrations of SSW (4%) (Fig. 10a). Any further increases in these concentrations led to a decrease in chitinase enzyme activity. Optimum enzyme yield could be possible with low pH at 8, 2.5% of inoculum size, and 225 rpm shaking speed (Fig. 10b). A similar response data are presented in Figs. (10a–c) revealed that chitinase activity was highly and interactively influenced by all selected variables. Figure (10c) showed the interaction of pH (B) and inoculum size (C) by SSW concentration (A) at the optimum value; the maximum chitinase activity was attained beyond a moderate level of inoculum size and low level of pH and any further increase of the previously mentioned factors didn't result in higher enzyme's activity.

### Chitinase partial purification with ammonium sulphate

The concentration of ammonium sulphate that was used in protein precipitation was varied in the range of 0–20%, 20–40%, 40–60%, and 60-80% (w/v). According to the ammonium sulphate fractionation, the maximum relative activity of chitinase was at the concentration of ammonium sulphate of 60–80% saturation. with specific activity of 48.13 related to the crude enzyme (Table 5).

**Table 5:** Partial purification of *P. megaterium* chitinase using ammonium sulphate method

Ammonium sulphate concentration	Activity (U/mL)	Protein (U/mg)	Specific activity	Relative activity %
20%	48.69	33.14	1.470	17.80
40%	150.2	14.55	10.32	54.88
60%	200.02	5.230	38.28	73.19
80%	260.0	4.369	59.51	95.13

### Cytotoxicity investigation

**Fig. 11:**
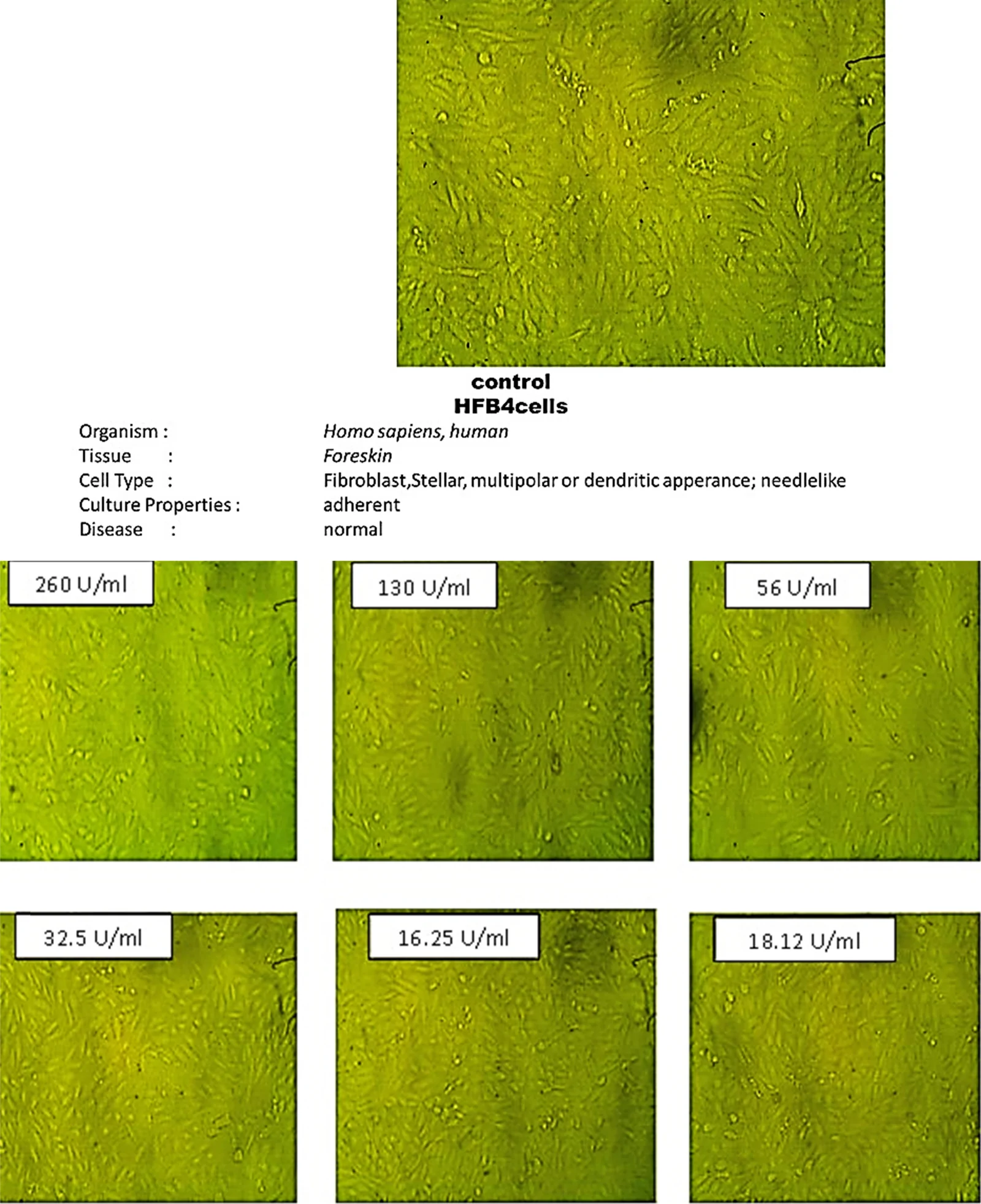
Microscopic images for skin normal cells viability influenced by various concentrations of partially purified chitinase

The cytotoxicity activity assay using skin normal cell lines (HEB4) revealed that the partially purified chitinase was safe in high concentrations up to 260.0 U/mL as shown in Fig. 11 and Table 6. The cell morphology of the skin cell lines over the different concentrations did not reveal any cell changes indicating the high biocompatibility.

**Table 6:** Cytotoxicity assessment for skin normal cells (HFB4) viability influenced by various concentrations of partially purified chitinase

ID	U/mL	O.D			Mean O.D	±SE	Viability %	Toxicity %	IC_50_ ± SD
HFB4	–	0.566	0.565	0.563	0.564000	0.002082	100	0	µg/mL
Chitinase	260	0.576	0.578	0.550	0.560677	0.001764	99.33303835	0.766961652	–
	130	0.564	0.567	0.560	0.564353	0.001453	99.64300815	0.353982301	
	56	0.596	0.520	0.553	0.553000	0.003000	99.78100390	0.235988204	
	32.5	0.516	0.512	0.561	0.566000	0.001732	99.78601470	0.176991155	
	16.25	0.565	0.523	0.560	0.563333	0.001202	99.81401186	0.117994143	
	8.12	0.561	0.554	0.559	0.563767	0.001764	99.98500514	0.117994133	

## Discussion

There is increasing concern to apply easy, large-scale production, cost-effective, and applicable techniques for industrial enzyme production. Microbial enzymes are considered a novel procedure for producing industrial enzymes using algae, fungi, and bacteria. By circumventing extreme pH and temperature ranges of industrial enzymes, microbial production has different benefits over chemical processes [3, 5, 35]. Chanworawit et al. [3]; Din et al. [36] reported that microbial methods have achieved perspectives through their strain selection, and optimization of cultivation conditions including nutritional factors, pH, temperature, incubation time, and volume of the biological material to be used in large-scale and commercial applications. Recently, bacterial industrial enzymes using various wastes and by-products have attracted researcher's attention for their incredible benefits such as ease of culturing, production of extracellular enzymes, and the simplest experimental conditions such as temperature, pH, and short generation time [3, 35].

In the current work, *B. amyloliquificiens*, *B. licheniformis*, *P. megaterium*, and *S. maritimus* thermophilic strains were investigated for chitinase fermentation on shrimp shell waste (SSW) broth for 48 h of incubation and 150 rpm shaking speed. Among all the tested bacteria *P. megaterium* (50.68 U/mL) was the most potent strain in chitinase activity (4.5, 3.33, 12.5-fold) and cell mass (2.83, 2.42, and 4.25-fold) compared to the other strains *B. licheniformis, B. amyloliquefaciens*, and *S. maritimus*, respectively.

As demonstrated by Veliz et al. [7]; Shoda [37]; Khan et al. [38]; Oyeleye and Normi [39], *Bacillus* sp. is a major source of bioactive natural chemicals that generate significant quantities of safe and advantageous secondary metabolites, such as chitinases. Numerous bacteria, including *Streptomyces* sp., *B. pumilus* [40], *B. subtilis* [41], *B. licheniformis* [42], exhibit chitinolytic properties. Additionally, after 48 h of incubation, Shalaby et al. [43] discovered that *B. licheniformis* and *B. subtilis* NRRL 1315 exert chitinase by 2.28 and 0.22 U/mL, respectively.

The development and metabolism of microorganisms depend heavily on the components of the fermentation medium. Therefore, medium optimization is a crucial step for maximizing yield and productivity as well as minimizing production costs [44]. Many microorganisms have been reported to be efficient in the decomposition of shrimp shell waste (SSW) [45]. The use of ammonium salts [46], yeast extract [47], is typical for the separation and degradation of shrimp shell detritus, along with additional nutritious additives. Sorokulova et al. [45] reported employing SSW as the only source of carbon and nitrogen without any additional supplementation, which is what happened in the current study. Due to that, the concentration of SSW is the key factor for chitinase fermentation using SSW medium without any supplementation as a unique source of carbon, nitrogen, and minerals. Therefore, SSW with different concentrations has been tested for chitinase optimization. The results demonstrated that maximum chitinase production with 5% SSW for *P. megaterium* with 50.86 U/mL. The CDW was estimated at 4.0, 2.2, 1.9, and 1.0 g/L for *P. megaterium*, with SSW concentrations of 5, 10, 15, and 20 g/L, respectively. Another study by Sorokulova et al. [45] utilized *B. cereus* 8–1 and *Exiguobacterium acetylicum* to decompose 3% shrimp shell waste broth. Also, Mejia-Saules et al. [46] used 6% shrimp shell powder broth for chitinase fermentation using *Serratia marcescens* WF.

In the current investigation it was obvious that the chitinase maximal production was for up to 72 h and before and after this incubation time the production was at low levels. According to Kuddus and Ahmad [10]; Hao et al. [48], in much submerged fermentation (SmF) processes, extending the incubation period after the product reaches its maximum yield can lower it because of metabolite degradation, nutrient deprivation, and the generation of hazardous chemicals in the medium, which inactivates the enzymes’ secretory machinery. In line with our observations chitinase fermentation at 72 h was the maximum (50.9 U/mL) according to Mejia-Saules et al. [46] using *S. marcescens* WF. This is in contrast to the results of Kuddus and Ahmad [10], who discovered that after 48 h of incubation, the chitinase production of *Aeromonas hydrophila* and *A. punctata* HS6 increased to reach 80.9 U/mL and 82.36 U/mL, respectively, and after that time the enzyme production in both strains progressively declined. However, after 96 h of fermentation, Sasi et al. [49] discovered that the *B. licheniformis* (SSCL-10) exhibited 3.4 U/mL of enzyme activity.

The incubation temperature adjustment affects the development of bacteria and the generation of enzymes because fermentation temperature affects several biological processes. According to the data currently available, 60° C was the ideal temperature for chitinase production, making it a viable option for challenging industrial settings. Garcia-Fraga et al. [50] demonstrated that the temperature at which *Haloarcula japonica* produced its chitinase was 40° C. While Fu et al. [51] observed the chitinase preferable temperature was a high temperature of 65° C with *Paenibacillus barengoltzii*. In contrast to all previous Mejia-Saules et al. [46] produced chitinase using *S. marcescens* WF at 28° C.

Also, it could be concluded as reported by Kuddus and Ahmad [10] and Pan et al. [52] that, the pH of the fermentation medium is crucial for the processes of cell development and metabolism. The optimum pH was observed at 7.0 and any pH elevation led to chitinase production reduction. In the same manner, Akeed et al. [2] discovered that chitinase activity was lowest at pH 4.0 and pH 9.0, and that the highest chitinase synthesis occurred at pH 7.0. Whereas Shanmugaiah et al. [53] demonstrated that the preferable pH for chitinase production was pH 8.0 for *B. laterosporous*.

The shaking submerged fermentation media (SmF) technique is a better method of producing chitinase than using static conditions, according to Akeed et al. [2]. The current investigation indicated that 200 rpm was the optimal shaking speed. Mejia-Saules et al. [46] used *S. marcescens* WF at a shaking speed of 180 rpm to produce chitinase. According to Gupta et al. [54] *Bacillus* sp. produced the most chitinase activity at 150 rpm. In a similar vein, Shivalee et al. [55] found that *B. licheniformis* B307 produced the most chitinase activity when it was shaked at a speed of 150 rpm, after which it dropped as the speed increased.

An essential component of metabolism and the effectiveness of chitinase synthesis is inoculum size. Any increase or decrease in the inoculum size volume had a substantial impact on the production of chitinase, with 2% being the most appropriate inoculum size. In parallel with the current findings, Mejia-Saules et al. [46] produced chitinase using 2% of *S. marcescens* WF inoculum. While Sorokulova et al. [45] produced chitinase using 10% inoculum of *B. cereus* 8–1 and *Exiguobacterium acetylicum*.

After determining the ideal levels of the factors affecting chitinase production, the second optimization level using response surface methodology (RSM) utilizes two optimization steps: Plackett–Burman in the first step and the central composite design in the second to determine the ideal level for each important factor. The Plackett–Burman screening experimental design collected data showed a wide variation of the response (chitinase activity) from 265.63 to 122.79 U/mL in the twelve different experiments due to the powerful influence of the variables on the activity of chitinase. With low levels (− 1 level) of 5.0% SSW concentration, inoculum size at 2%, and incubation periods of 48 h, and high levels (+ 1 level) of incubation temperature at 60° C, pH 9.0, and shaking speed of 200 rpm , the first run (number 1) had the highest chitinase activity. Chitinase activity was lowest in run 11 when the SSW concentration was low (− 1 level) at 5.0% and the pH was 7.0. The incubation temperature was high (+ 1 level) at 60° C, the shaking speed was 200 rpm, the inoculum size was 3%, and the incubation duration was 72 h. This may be because the fermentation medium's pH level and/or inoculum size prevent *P. megaterium* from growing properly, which in turn prevents the formation of chitinase.

From the CCD experimental design, the interactive production of chitinase was influenced by all selected variables. When SSW% is maintained curve, the maximum chitinase activity was attained beyond a moderate level of inoculum size (2.5%), pH 8, and shaking speed (225 rpm). And any further increase in inoculum size, pH, and shaking speed didn’t result in elevating enzyme activity.

Mejia-Saules et al. [46] conducted a similar study on chitinase optimization production, employing *S. marcescens* WF to produce chitinase. They used an experimental design with a surface response model to numerically assess the impact of pH, incubation temperature, and dry crude shrimp shell content (CSS). The chitinase response (Y) was represented by a second-order polynomial equation of crude shrimp shells, pH, and temperature-tested variables while the temperature was still a constant factor. A 6% crude shrimp shell, pH 6.5, and a fermentation temperature of 28° C for up to 72 h were the outcomes of the chitinase production optimization procedure. Toxicity investigation is a crucial issue for any enzyme to be applicable in many sectors, such as agriculture, pharmacy, cosmetics, and detergent sectors. In this line, our study exhibited no toxicity concerns when applied to HFB4 skin cell lines. In contrary to the current findings, Silano et al. [56] observed that the chitinase from *Streptomyces violaceoruber* (strain pChi) revealed oral toxicity issues at a dose of 791 mg when applied to rats feeding routine.

## Conclusion

This study demonstrated that shrimp shell waste can be effectively utilized as a substrate for microbial chitinase production. Among the four tested strains, *Priestia megaterium* AMD 2024 exhibited the highest chitinolytic activity. Optimization using one-variable-at-a-time (OVAT) identified suitable baseline conditions, while response surface methodology (RSM) significantly enhanced enzyme yield, achieving 273.3 U/mL—a 2.93-fold increase compared to OVAT. Partial purification via ammonium sulphate saturation (60–80%) resulted in 260.0 U/mL activity with 95.13% relative activity. Cytotoxicity assays confirmed the biocompatibility of the purified enzyme with HFB4 normal skin cells, supporting its safe application. These findings establish *P. megaterium* AMD 2024 as a promising candidate for eco-friendly chitinase production and highlight shrimp shell waste as a valuable raw material for sustainable bioprocessing. The optimized enzyme can be applied in agriculture, industry, and cosmetics, contributing to waste valorization and circular economy practices.

## Data Availability

‘‘The datasets generated during the current study are available in the [NCBI] repository: 1-*Streptomyces maritimus* MSQ-20219 Accession number: LC638499 https://www.ncbi.nlm.nih.gov/nuccore/LC638499.1 2-*Bacillus amyloliquefaciens* strain BT 2022 Accession number: OR251122 https://www.ncbi.nlm.nih.gov/nuccore/OR251122 3-*Bacillus licheniformis* strain Basma87 Accession number: OP547873 https://www.ncbi.nlm.nih.gov/nuccore/OP547873 4-*Priestia megaterium* strain AMD 2024 Accession number: PP528505 https://www.ncbi.nlm.nih.gov/nuccore/PP528505’’.
